# 

**Published:** 1904-02-27

**Authors:** 


### CONTENTS.

**The Infectivity of Enteric Fever** - - - - - - 333

**A University Diploma in Tropical Medicine** - 334

**Annotations** - - - - - - - - - - - - - 335

**Notes and News** - - - - - - - - - - - 335

**Hospital Clinics and Medical Progress—**

The Sanatorium Treatment of Consumption - - - - 387

The Cardiac Muscle - - - - - - - - - 33[illegible]

Progress in Fevers - - - - - - - - - - 39[illegible]

Progress in Diseases of the Blood - - - - - - - 392

Progress in Diseases of the Heart - - - - - - 393

**Hospital Administration—**

The Hospitals Association - - - - - - - - 394

Hospital Meetings - - - - - - - - - - - 396

**Editor's Letter-Box** - - - - - - - - - 397

**The Student of Medicine** - - - - - - - - 398

*NURSING SECTION.*

**Notes on News from the Nursing World—**

The King's Inspection of Osborne House—Queen Alexandra's Military Nursing Service—The Case of "Ragging" at an Asylum—The Infirmary Nurse and the "Useful Inmate"—Dental Nurses—Irish Hospitals and the Midwives Act—The Asylums Board and State Registration—The Nurses of the Royal Devon Hospital—New Matron of Leydsdorp Hospital—The Problem at Oldham—Progress at Huddersfield Union Infirmary—A Nurse killed while Cycling—"A Hard Case"—Resignation of Nurses at an Irish Infirmary—The Uncertificated Midwife at Work—The Nursing Staff at the Ringworm Schools—Short Items - - - - 293-296

**The Nursing Outlook** - - - - - - - - - 297

**Lectures upon the Nursing of Mental Diseases** 298

**How Nurses are Manufactured in France** - - 299

**Everybody's Opinion** - - - - - - - - 301

**Novelties for Nurses** - - - - - - - - - 301

**Where to Go** - - - - - - - - - - - 301

**Echoes from the Outside World** - - - - - - 302

**A Book and its Story** - - - - - - - - - 303

**Notes and Queries** - - - - - - - - - - 304

**For Reading to the Sick** - - - - - - - - - 304

